# Her Heart's Mistake

**Published:** 1889-05-25

**Authors:** E. D. Cumming

**Affiliations:** Author of "An Ordeal by Silence"


### Her Heart's Mistake.

By E. D. Cumming, Author of "An Ordeal by Silence."

*(Continued from page 109.)*

### Chapter VII.—A Sick Room.

George Bairdsley passed a wakeful, restless night, and the dawn found him tossing about on his bed in a condition of mind and body which seemed to have made another man of him. Only those who have had it, and retain a vivid recollection of their first "fever" in India, can understand the physical and mental misery which possessed George Bairdsley the morning after his accident on the race-course.

"Just what I expected last night!" said Dr. Harrison, when he came in to see his patient-visitor. "I ought to have insisted upon your taking that quinine, whether you wanted it or not. Now you have got a sharp attack of fever from the pain yesterday."

Bairdsley had fought stoutly against being treated as an invalid when he was laid in bed the previous evening. He had been stunned in the hunting-field and elsewhere often before, and did not think anything of a sprained wrist. Why couldn't the Doctor let him stay up and have his dinner? He acknowledged that he felt a little shaky, but that was nothing. If he was not quite himself next morning, he would obey Dr. Harrison's orders and remain in bed. So, with many unspoken reflections upon the foolhardiness of young men who won't believe that sprains and dislocations in India are not to be treated so lightly as the same mishaps at home, the Doctor unwillingly allowed George Bairdsley to have his own way; and fever was the result.

"How long will it last?" he enquired.

"Not many days, we must hope," replied Dr. Harrison, placing his clinical thermometer under the patient's arm.

"What's the figure?" asked Bairdsley, as the Doctor examined the thermometer.

"A hundred and two. Higher than I thought. Lie still, and keep yourself quiet. I'll bring you the mixture when I come back from the hospital."

"There's nothing infectious about this abominable fever, is there?"

"O dear no; you will be up and about by Sunday, I hope. Don't be alarmed about yourself. I know well how a fellow feels when he gets his first go of it."

"I suppose I need not tell you not to smoke," he added, with a smile, as he turned to leave the room.

It was hardly necessary, for Bairdsley felt that, ill as he was, he could rise up and kill anyone who came near him with the faintest reminiscence of tobacco on his person, and he only shook his head in reply. It was half-past six when Kate asked from behind the purdah whether she might come in.

"This is a bore, Kitty," he remarked, ruefully, as she sat down at his bedside.

"I can't forgive myself for it, George," said poor Kate, who had been reproaching herself ever since her lover's fall for her eagerness to have him ride Baroness. "I had a presentiment yesterday when you mounted that something was going to happen. The mare was so excited."

"Why, bless you, dear, it wasn't her fault. Arkwright ought to have had the fall for hustling Volunteer as he did, instead of letting the horse take his own pace at the hurdle. The sticks he kicked out got between Baroness's legs as she landed; that was what brought her down. Is she damaged?"

"No, nothing to speak of. I wish she had been hurt instead of you."

"I wonder she didn't break her neck."

"She escaped with a little hair and skin rubbed off her face and shoulder, and a bruise on the leg. Ram Buksh says she trots quite sound this morning."

"That's right. We'll put her in training for the big autumn meeting, and I shall be surprised if we don't pull off a race or two with her."

"Indeed, George, you are never going to ride a jump-race again—never. Those are my orders."

Mrs. Brent's appearance put a stop to the affectionate dispute to which this prohibition gave rise. She came in overflowing with speeches of condolence and suggestions of food, with which she harassed the invalid until he could hardly restrain signs of impatience. It was only with strangers that the Doctor's sister was silent and reserved; but George had become almost a member of the family. He had won her heart by his polite attentions to her, and now he suffered for it.

"Won't you try and eat a little jelly if I make it for you, George? There's nothing like jelly when one has fever, I think. Or a milk pudding? Custard or something of that kind. Our bobachee makes better light puddings than any other cook I have ever met, and it is the very best thing for you. It's so cooling and easy to eat. Or, stay; could you try a mango? I always think fruit is the best thing to eat when one has fever. I always have a longing for fruit when I have fever. I remember when I had my first attack we were at Barrackpore, close to Calcutta, a horridly feverish place. I ate nothing for over seventy hours, and then I had eleven plaintains; my poor Charley counted them. Bananas, they call them in England. Shall I send your khit to the bazaar to get a few? If you would only say what you would like, you shall have it at once."

But Bairdsley declined jelly, custard, and fruit, and tea in turn, and, recognising that he must ask for something, requested a lime and soda, which he did not want, but which looked a genuine and natural requirement.

"I hope your dear aunt is not going to help nurse me, Kitty," he said, when Mrs. Brent had left the room to prepare the drink for him herself. "If she does, I sha'n't want nursing long."

"She means well, George; she is the kindest creature on earth."

"I give her all credit for her good intentions," said Bairdsley with some irritation. "But I only hope she won't insist upon nursing me."

Kate did not answer, but rose and went to the door to call the punkah-wallah; for the sun was getting high and the windless morning was close and hot. The bearer removed the mosquito curtain which had covered the bed during the night, and, under Kate's directions, pinned thick towels on to the punkah fringe to increase its efficiency.

"It's fortunate that the rainy season is so near, George," she remarked, as she went on with her arrangements for his comfort; "but we must do our best to keep the room cool while this heat lasts."

Bairdsley slept a little that night, but awoke without any decrease in his temperature, and with as little appetite as he had had the morning before. Kate carried her work into his room, and sat with him nearly all day, reading and talking to him, and using all her influence to make him carry out her father's medical instructions. He certainly was a very trying charge, and even the devoted Kate found herself framing excuses for the behaviour of her hero. His restless irritation and unreasoning selfishness astonished and pained her. She hardly knew him to be the same man as the George Bairdsley of yesterday, who underwent the painful operation on the racecourse without a word. A fly hovering about his pillow provoked an outburst of bad language, directed at the servant who stood waving a huge palmleaf fan in place of the towel-fringed punkah, which had been stopped, as it made his head ache. He was barely civil to Mrs. Brent when she came to sit with them, and who, forewarned by Kate, ventured a remark only once in a quarter of an hour.

"My dear George, do let papa's bearer take your man's place for a few hours," said the young lady, as the servant responded for the hundredth time to his master's call for some trifle or other; "the poor wretch has been up two nights, and you never give him a minute's rest."

"*He* doesn't want rest," replied Bairdsley. "Here! go over to my bungalow and bring a clean handkerchief."

The heavy-eyed bearer, who had snatched about half an hour's sleep during each of the previous nights, went silently on his errand, and his master explained that these fellows were only too anxious to shirk their business when their employer was unable to look after them.

"You don't do them justice there, George," said Kate. "I can assure you that if a man treats his servants well, they are untiring in their care of him when he falls ill. You're not very kind to your man, dear, but you know that he has never left the verandah since Thursday evening, except for ten minutes now and then to get his food."

"It's what I pay him for," said Bairdsley, "and he always manages to be at his food when I want him."

"Well, let papa's bearer wait on you for a little so that your's can get a few hours sleep and be fresh to-night."

"It's very good of you, Kate, but the Doctor's man can't speak English. The other fellow is all right; don't bother yourself about him."

And Kate, whose tender heart could not bear to see any living creature misused, but who feared to anger Bairdsley now while he was ill, let the subject drop, and tried to save the unfortunate bearer by anticipating the invalid's wants herself; no easy task, when they were for the most part imaginary.

The Sunday, which Dr. Harrison had hoped would mark the subsidence of the fever, came and went, but Bairdsley was no better. He had forced himself to take a little soup and toast occasionally, but could not look at anything more substantial, and the heavy wearing pain was dulled by a sense of weakness. Kate spent nearly all her waking hours by his side, taxing her ingenuity to ease him, and keep his mind occupied. He was consistent in his thoughtfulness for her, and insisted firmly upon her taking her customary ride and drive in the morning and evening. She must go, he said; she would see Captain Phelps or some of the other men at the tennis court or the club, and might ask one of them to come in and see him. She must take exercise if it was only to please him, and he should like her to take Mrs. Brent with her. He didn't want to say anything against Kate's aunt, but she was more than he could stand in his present state. And Miss Harrison, who would not hear that kind-hearted woman "run down" even by George Bairdsley, affected to laugh at him, and promised to make her go also.

But the laugh died from her lips as she crossed the threshold of his room. Under the test of illness, her hero was dwindling away into an ordinary and not too amiable specimen of humanity. She had pictured a manly sufferer who would bear his troubles in enduring silence, and make light of the temporary restraints of a sick-room. A man whom it would be a pleasure and privilege for any woman to nurse and tend; a patient who would rise superior to his malady with high-bred courage, and disclaim the need of pity. She could not hide from herself the disappointment she felt at the common-place reality. He was peevish and nerveless; irritated by the smallest trifles, and ever ready to vent his irrational wrath upon the long-suffering servants. He let his pain master him, moaned about it incessantly, and showed a narrow-minded selfishness which was out of all keeping with the calm, lofty bearing Kate had worshipped in the man of a few days ago. Could this be his real character that she saw now? Was it possible that he had cast off his old manner with his clothes, and was now revealing himself in his true colours? Her heart sank as she asked herself these questions. And then she indignantly turned upon herself, and replied to them with all the warmth of her generous nature by asking others. Was this the time to judge any man's character? Was it fair to expect that he should maintain the unruffled front of a man in health now, while his head throbbed unceasingly, while he was in high fever, while he had to bear the pain of such injuries as he had sustained in that fearful fall. Was it kind of her to read him by the light of illness, the consequence of his devotion to her, let her remember? The man did not exist who could ignore such torments. She had never supposed that George was a perfect being who could lie day after day in semi-darkness without a murmur, tolerating the stupid mistakes of native servants with indomitable sweetness and patience. He would become his old self again when this horrid fever subsided, and banish these unworthy doubts of hers.

She dressed and went out to drive with her aunt, overcoming that good lady's scruples about leaving poor dear George alone, by assuring her that he was trying to go to sleep. She wanted to see some of his friends and ask them to call and brighten him up a little. Men were so terribly selfish when their friends were ill; they always seemed uneasy and uncomfortable in a sick-room, and anxious to escape from it. Colonel Tenterdale had come in twice, and remained about a quarter of an hour each time. Captain Phelps certainly had come in every day, and he was the only man George cared particularly to see. But he was working hard now and could not make very long calls. Little Wentworth had paid him a visit, but he was so stupid and asked such silly questions that she had not been surprised when the invalid snubbed him. Then Major Arkwright had been to see him, and had annoyed George by endeavouring to demonstrate that he had himself to blame for his fall by letting Baroness take off so close to Volunteer—which was sheer nonsense on the face of it, and enough to irritate anybody. If the Major did think he had been in the wrong it was not nice of him to come for the express purpose of telling him so; and when that gentleman came up to the carriage to ask how the patient was progressing, she gave him a "bit of her mind"—a proceeding which only served to raise the delinquent's high opinion of her, and caused him to mentally congratulate Bairdsley on his good luck in securing a girl with such boundless belief in him. When *he* came home from his pig-sticking excursions with wounds incurred by a fall with his horse, Mrs. Arkwright would hear his explanations, but invariably suggested how he might avoid these troubles if he would simply keep his eyes open, and look where he was going.

The officers of the 50th Punjaub infantry were giving a little impromptu dance that evening, and Kate had, as a matter of course, been invited to it. Mrs. Arkwright, being the "senior lady" of the regiment, was acting as hostess, and she came to Miss Harrison to have a little chat about the programme.

"I don't think I shall come this evening, Mrs. Arkwright," said Kate, in answer to a request that she would put in an appearance early. "I don't want to leave George alone."

"Now, Kate, we know how you are treating that young man. You are simply spoiling him by too much care and kindness. If you go on like this he won't let you out of his sight, and you will find that awkward and likely to give rise to misunderstandings—to put it mildly. I won't go away until you promise to come to-night, for at least an hour or two."

Kate would give no promise: she loved dancing, but could not hope to enjoy a waltz, weighed down as she was with the thought of her invalid lying in the dark at home, and Mrs. Arkwright had to give up pressing her. She waylaid Mrs. Brent, however, and having easily wrung from her an admission that the dance would "do her niece good," further extorted an undertaking to acquaint George Bairdsley with the fact that the 50th had issued invitations for an "at home" that evening.

"I don't believe he will allow her to miss it," said Mrs. Arkwright, "though Jack told me that he was as savage as a bear when he went to see him. It wouldn't be like Captain Bairdsley to want her to stay at home; but if he does, the man's a brute and nothing else."

Mrs. Arkwright was somewhat inclined to indulge in strong expressions, and the bare notion of her beloved Kate being deprived of a dance because her "young man" had fever was intolerable.

"Kitty," said Bairdsley, when she came into his room that evening, "why didn't you tell me that the 50th were giving a dance to-night?"

"I didn't think of it," fibbed Miss Harrison.

"Well, you will oblige me by thinking of it now. Go and get ready, unless you dress for these affairs after dinner."

"I don't want to go to-night, George, dear; I'd rather stay with you."

"You must go. I'm better to-night, and won't have you stay away."

"I prefer to stay at home."

"Now, Kitty, you are to go. I sha'n't let you confine yourself to the house on my account, and you will please me if you go to-night."

He was so obviously in earnest that Kate gave way, and consented to go for an hour, though she would, as she said, have rather remained at his side. And she went to her own apartments, remorsefully thinking of her doubts that afternoon, when she told herself that his irritation and selfishness had disappointed her. He was a little better this evening, and look how changed he was; she had done him injustice in her thoughts, and was more than ever convinced of his nobility of character and superiority to other men. She was proud to own his authority, and obey him.

But the punkah-wallah, crouching, rope in hand, against the wall of George Bairdsley's room, under a continuous volley of threats and abuse, was of a different opinion; and the bearer sympathised with the punkah-wallah, low caste man though he was.

*(To be continued.)*

